# A rare case of primary mesenteric gastrointestinal stromal tumor with metastasis to the cervix uteri

**DOI:** 10.1186/1477-7819-5-137

**Published:** 2007-11-29

**Authors:** Nupur Gupta, Suneeta Mittal, Neena Lal, Renu Misra, Lalit Kumar, Sunita Bhalla

**Affiliations:** 1 Department of Obstetrics & Gynaecology, All India Institute of Medical Sciences, New Delhi, India; 2 Department of Obstetrics & Gynaecology, Moolcahnd Khairati Hospital, New Delhi, India; 3 Department of Medical Oncology, All India Institute of Medical Sciences, New Delhi, India; 4 Department of Pathology, Sir Gangaram Hospital, New Delhi, India

## Abstract

**Background:**

Gastrointestinal stromal tumors are CD117 (C Kit) positive mesenchymal neoplasms, that may arise anywhere in the gastrointestinal tract. Their current therapy is imatinib mesylate before or after surgery.

**Case presentation:**

We describe a case of 17-year-old female with metastasis to the cervix uteri of a primary mesenteric gastrointestinal tumor.

**Conclusion:**

Surgery remains the mainstay of known curative treatment. The manifestations of GIST are not restricted to the typical locations within the bowel; may have very unusual metastatic sites or infiltrations per continuitatem.

## Background

Gastrointestinal stromal tumor (GIST) is a rare mesenchymal tumor of the gastrointestinal tract with an incidence of 10–20 cases per million populations of which almost one third are deemed malignant [1]. We report this rare case of primary mesenteric tumor metastatic to the cervix that was diagnosed to be a GIST on histopathological examination.

## Case presentation

**Figure 1:**
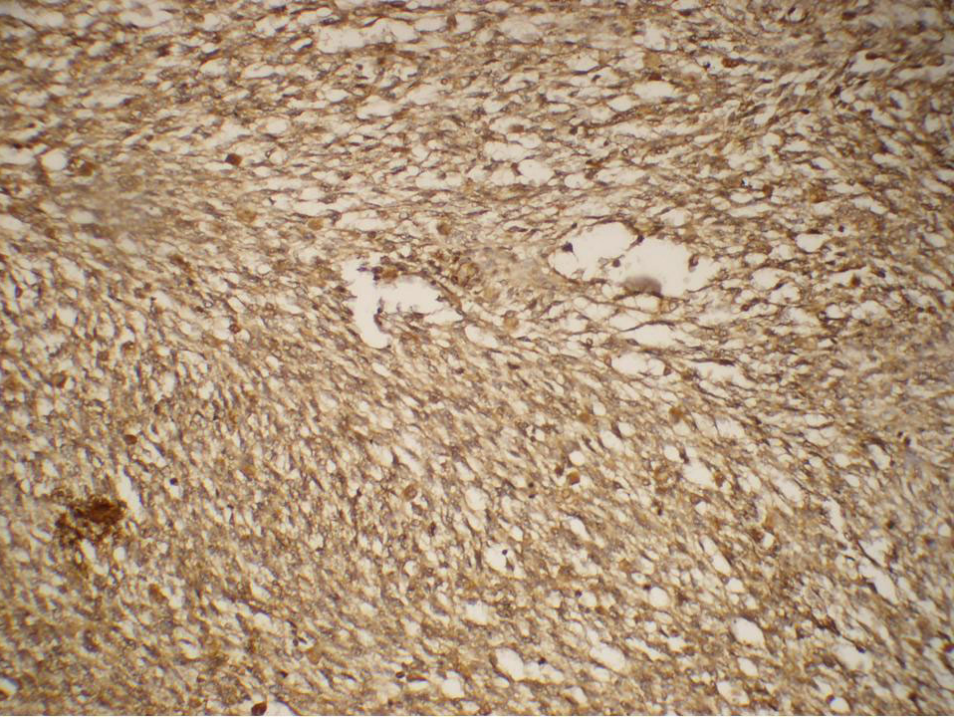
Cervix with metastatic gastrointestinal tumor, showing fascicles of spindle cells positive for CD 117 stain.

**Figure 2:**
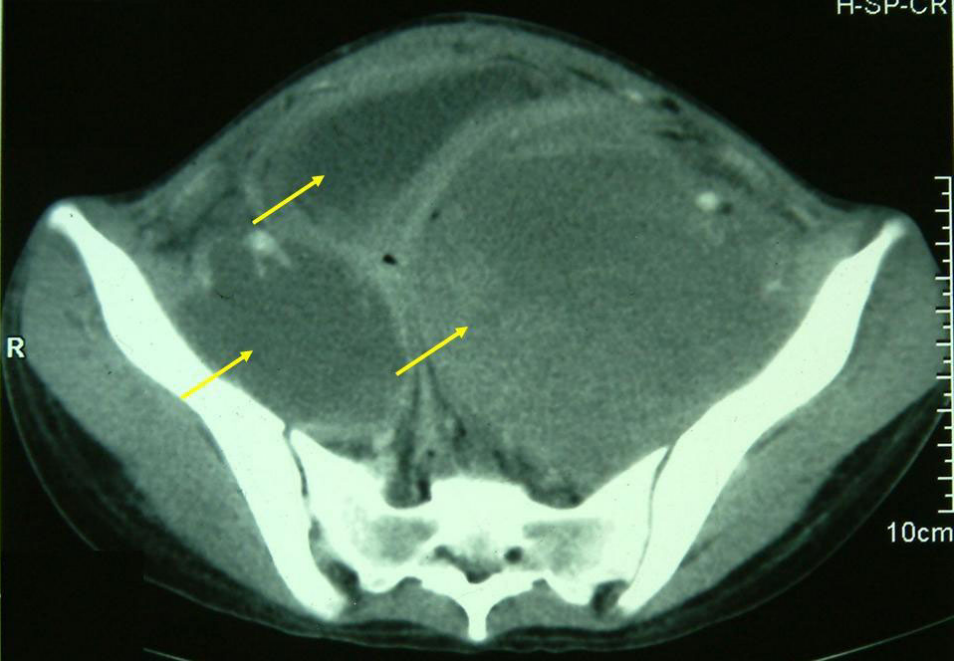
CT scan showing the tumor extensions.

A 17 year old unmarried female presented with history of menorrhagia and passing tissue per vagina since 3 months, low grade fever since 1 month and feeling of mass per abdomen since 10 days. On examination, she had a mass corresponding to 24 weeks size gravid uterus arising from pelvis, which was tender on palpation. There was anemia. Four units of blood transfusion were given in a private clinic. Her routine liver and renal function tests and coagulation profile were normal. Ultrasound followed by contrast enhanced computerized tomography (CT) showed bilateral adnexal masses with a large soft tissue heterogeneous mass suggestive of sarcoma of the uterus and presence of enlarged retroperitoneal lymph nodes. Examination under anesthesia revealed the same mass with side to side restricted mobility, and a fleshy, friable and vascular growth protruding into vagina. Biopsy from the growth was initially misdiagnosed as a leiomyosarcoma. Exploratory laparotomy was carried out as the patient was continuously bleeding and staging was performed. Intraoperatively, there was a large 30 × 30 × 12 cm friable mass with necrosis and hemorrhagic areas arising from the mesentery of the transverse colon, with tumor deposits were present all over the pelvic peritoneum. A solid right adnexal mass, 10 × 4 cm was also present. Resection of tumor with wide margin along with panhysterectomy and infracolic omentectomy was done. Cut section of uterus, with bilateral fallopian tubes and ovaries showed serosal deposits. Endocervix revealed a 4.5 × 4 × 1 cm cauliflower like, exophytic, polypoidal vascular friable growth lying at the lower end. Vagina was grossly looking normal. Histopathological examination revealed a primary gastrointestinal tumor of mesentery with >5 mitoses per 50 high power field and atypical spindle cells arranged in fascicles. There were metastasis in the cervix, serosa and omentum. Immunohistochemical stains showed positivity for vimentin, smooth muscle actin, desmin and CD 117 (Figure 1) and were negative for cytokeratin, epithelial membrane antigen and CD 34. Molecular genetic analysis (KIT mutation analysis) was not done due to its unavailability at our institute. Postoperatively, she received 6 units of packed RBC, fresh frozen plasma and platelet rich plasma each. She had a stormy postoperative period with fever, severe pain abdomen and developed a recurrence after 4 weeks with huge mass per abdomen and faecal fistula from the surgical scar. On computerized tomography, there was a large heterogeneous mass lesion in pelvis with areas of necrosis infiltrating sigmoid, iliopsoas with encasement of ureters causing bilateral hydroureteronephrosis. The mass was involving the iliopsoas muscle on left side and encasing the ureters bilaterally (Figure 2). Imatinib 400 mg qd was started which showed a minimal improvement in her clinical condition. She continued to have a huge pelvic mass with pain, fever, urinary and bowel symptoms for which palliative care was administered. RECIST (Response Evaluation Criteria in evaluating Solid Tumors) criteria were applied for response assessment. At 4 weeks, only 16% of the lesions achieved partial response (PR) by RECIST and 24% increased in size by a mean of 22.5% (progressive disease, PD).

## Discussion

GIST may present anywhere in the gastrointestinal tract, omentum, or mesentery. The most common sites are the stomach (60%), small intestine (15%), colon and rectum (5%), other abdominal organs including mesentery and omentum (5%) [2]. Usual age of presentation is around 60 years, predominantly seen in Caucasians with little sex difference. Pediatric GISTs have also been reported with predilection for girls. They have a multicentric distribution with a high risk of local recurrence and regional lymph node metastasis, show a predominantly epitheloid morphology and lack KIT mutations [3]. Patients with GIST usually present with abdominal mass, gastrointestinal haemorrhage, pain, nausea, and vomiting and/or weight loss. Our patient had an unusual clinical presentation of menorrhagia and passage of tissue per vaginum. The tumor is thought to arise from the interstitial cells of Cajal. On microscopy, 70% of GISTs are spindle cell tumors. Nearly all are immunohistologically positive to CD117 (KIT tyrosine kinase) as in our case, which differentiates it from true leiomyomas, neurofibroma, leiomyosarcoma and schwannoma [4]. A diagnosis of GIST was made in our patient on the basis of histological characteristics of the tumor, clinical presentation and immunohistochemical profile, including a positive test for the CD117 marker. Most of the GISTs result from a somatic mutation although rarely familial cases are reported. A KIT activating mutation occurs in 70 – 80%. There are settings, when mutation analysis might help with decision making and the decision for neoadjuvant treatment becomes easier when exon 11 mutation would be present than no mutation at all. Primary treatment is surgical resection of the tumor. Induction treatment with imatinib (neoadjuvant chemotherapy) should be considered before major radical surgery, if histology can be obtained before as possibly mutilitating surgical procedures can be avoided. Given the high probability of response, it may also be considered in unresectable or inoperable metastasis as it improves the surgical operability and morbidity and successfully reduces tumor size in GISTs [5].

This is usually followed by relapse in more than 50% of patients, which is mostly intraabdominal. Targeted adjuvant therapy against KIT tyrosine kinase using imatinib mesylate (ST1571) 400 mg has revolutionized the treatment of GISTs [6]. GISTs have been the lead example, that classical response criteria are insufficient to measure "clinical success." All large trials have shown that NC and PR responses show equally good response durations and no significant difference in the effect on overall survival. This had a huge impact on many trials, not only GIST, to use progression free survival as a primary endpoint, instead of response. GIST may lose KIT expression after imatinib treatment and require a thorough examination of the parts of the resected specimen. Some centres perform monthly CT for response assessment until tumor progression. Sometimes, false positive complete response (CR) is noted on PET CT. Parameters recorded are largest dimension, new lesions, and any new features and tumor response is assessed through RECIST criteria [7]. They are a new set of tumor response criteria adopted by WHO, the National Cancer Institute and the European Organisation for Research and Treatment of Cancer. To our knowledge, this is the first case report of mesenteric GIST with metastasis to the uterine cervix in literature. GIST may also present as a pelvic mass [8] or metastasize to the ovary [9-11].

## Conclusion

Surgery remains the mainstay of known curative treatment. Recently, the diagnosis and management of GIST has undergone a revolution with the emergence of CD117 staining and the transforming oncogene (KIT mutation). Its specific targeted inhibition is greatly effective in treating GIST. The manifestations of GIST are not restricted to the typical locations within the bowel; may have very unusual metastatic sites or infiltrations per continuitatem. Thus, a multidisciplinary approach including a gynecologist and a medical oncologist can improve the prognosis of patients with GIST.

## Competing interests

The author(s) declare that they have no competing interests.

## Authors' contributions

NG, literature search prepared the draft manuscript, SM, NL, RM helped in preparation of manuscript, LK, helped in preparation of oncology part of the manuscript, SB contributed the pathological aspect of case. All authors read and approved the final manuscript.
